# Corticosteroids in septic shock: a systematic review and network meta-analysis

**DOI:** 10.1186/s13054-017-1659-4

**Published:** 2017-03-28

**Authors:** Ben Gibbison, José A. López-López, Julian P. T. Higgins, Tom Miller, Gianni D. Angelini, Stafford L. Lightman, Djillali Annane

**Affiliations:** 1 Cardiac Anaesthesia and Intensive Care, Bristol Heart Institute – University Hospitals Bristol NHS Foundation Trust, University of Bristol, Bristol, UK; 2 School of Social and Community Medicine, University of Bristol, Bristol, UK; 3 Centre for Research Synthesis and Decision Analysis, School of Social and Community Medicine, University of Bristol, Bristol, UK; 4 Cardiac Surgery, Bristol Heart Institute – University Hospitals Bristol NHS Foundation Trust, University of Bristol, Bristol, UK; 5 Henry Wellcome Laboratories for Integrative Neuroscience and Metabolism, School of Clinical Sciences, University of Bristol, Bristol, UK; 6 Medicine: Critical Care Medicine, Hôpital Raymond Poincaré, Assistance Publique Hôpitaux de Paris (APHP), Garches, France; 7 School of Medicine, Université de Versailles Saint-Quentin-en-Yvelines, Versailles, France

**Keywords:** Sepsis, Critical care, Glucocorticoids, Steroids, Adrenal, Septic shock

## Abstract

**Background:**

Multiple corticosteroids and treatment regimens have been used as adjuncts in the treatment of septic shock. Qualitative and quantitative differences exist at cellular and tissular levels between the different drugs and their patterns of delivery. The objective of this study was to elucidate any differences between the drugs and their treatment regimens regarding outcomes for corticosteroid use in adult patients with septic shock.

**Methods:**

Network meta-analysis of the data used for the recently conducted Cochrane review was performed. Studies that included children and were designed to assess respiratory function in pneumonia and acute respiratory distress syndrome, as well as cross-over studies, were excluded. Network plots were created for each outcome, and all analyses were conducted using a frequentist approach assuming a random-effects model.

**Results:**

Complete data from 22 studies and partial data from 1 study were included. Network meta-analysis provided no clear evidence that any intervention or treatment regimen is better than any other across the spectrum of outcomes. There was strong evidence of differential efficacy in only one area: shock reversal. Hydrocortisone boluses and infusions were more likely than methylprednisolone boluses and placebo to result in shock reversal.

**Conclusions:**

There was no clear evidence that any one corticosteroid drug or treatment regimen is more likely to be effective in reducing mortality or reducing the incidence of gastrointestinal bleeding or superinfection in septic shock. Hydrocortisone delivered as a bolus or as an infusion was more likely than placebo and methylprednisolone to result in shock reversal.

**Electronic supplementary material:**

The online version of this article (doi:10.1186/s13054-017-1659-4) contains supplementary material, which is available to authorized users.

## Background

The place of therapeutic corticosteroids in critically ill patients with sepsis is controversial. Two main questions still exist in this population. First, is there a group of critically ill patients who are relatively deficient in corticosteroids, and if so, how they should they be treated [1]? Second, do steroids given to all critically ill patients improve outcomes [2]? These questions have been investigated primarily in those patients with septic shock, and sufficient studies have been conducted to allow multiple meta-analyses [2–4], including a recently updated Cochrane review [2]. However, not all therapeutic corticosteroids are the same. Even at dose equivalency, some corticosteroids have more immunosuppressive properties (e.g., dexamethasone), and some have more mineralocorticoid and vasoreactive properties (e.g., hydrocortisone) [5]. This, tied with the evidence that endogenous glucocorticoids are secreted in a pulsatile manner in health [6], major surgery [7] and critical illness [8] warranted further analysis of the effects of the individual drugs and the dose regimens used. We performed a network meta-analysis (NMA) on the data used for the Cochrane review to establish the likely effectiveness of each drug and therapeutic regimen in adults with septic shock.

## Methods

Inclusion criteria for the Cochrane review [2] were as follows:

- Randomised controlled trials with and without blinding
- Children and adults with sepsis defined by the following:
  - Documented infection, defined as culture or Gram stain of blood, sputum, urine or normally sterile body fluid that is positive for a pathogenic micro-organism, or a focus of infection identified by visual inspection (e.g., ruptured bowel with the presence of free air or bowel contents in the abdomen found at the time of surgery, wound with purulent drainage); and
  - At least two symptoms of a systemic inflammatory response syndrome, such as fever (body temperature >38 °C) or hypothermia (<36 °C), tachycardia (>90 beats per minute), tachypnoea (>20 breaths per minute) or hyperventilation (arterial carbon dioxide tension <32 mmHg) and abnormal white blood cell count (>12,000 cells/ml or <4000 cells/ml) or >10% immature band of neutrophils; and
  - At least one sign of organ dysfunction, that is, metabolic acidosis, arterial hypoxaemia (arterial oxygen tension/fraction of inspired oxygen <250 mmHg), oliguria (<30 ml/h for ≥3 h), coagulopathy or encephalopathy; and
  - Septic shock defined by a combination of these criteria and the presence of hypotension (persisting systolic arterial pressure <90 mmHg) that is refractory to fluid resuscitation and requires vasopressor support, that is, >5 μg/kg of body weight per minute of dopamine or any dose of epinephrine or norepinephrine.
- Interventions were regarded as systemic treatment with any type of corticosteroid preparation, whereas controls were standard therapy or placebo.

Data from trials of acute respiratory distress syndrome (ARDS) were included when separate data were available for participants with sepsis or when contact with study authors resulted in provision of the data.

Studies that were included for analysis in the Cochrane review were initially included in this analysis. However, we excluded all data from children (<18 years), as well as those studies that were designed to investigate the effect of corticosteroids on respiratory function in ARDS and pneumonia (primary outcome measures were purely respiratory function). Cross-over studies where both groups received steroids and no information was published on outcomes at the cross-over were also excluded. The RevMan 5 file for the published meta-analysis was provided by the lead author of the Cochrane review [2]. Full details of the methods of data search, abstraction, handling and assessment are available from http://onlinelibrary.wiley.com/doi/10.1002/14651858.CD002243.pub3/full.

### Outcome measures

Interventions were grouped as follows:

- Hydrocortisone infusion
- Hydrocortisone bolus
- Dexamethasone bolus
- Methylprednisolone bolus
- Methylprednisolone infusion
- Prednisolone

Infusions were defined as treatment regimens where there was continuous delivery of a drug without interruption until the end of the treatment period. The bolus group was defined as those receiving treatment regimens with planned temporal interruptions to corticosteroid drug delivery.

Outcomes were grouped as follows:

- Up to 28-day mortality
- Hospital mortality
- Intensive care unit (ICU) length of stay (LoS)
- ICU mortality
- Shock reversal
- Incidence of gastrointestinal (GI) bleeding
- Incidence of superinfections

Hyperglycaemia was a commonly reported negative outcome of the use of glucocorticoids. However, the definition of hyperglycaemia between studies varied so widely that it was not possible to group the results.

### Data management

One author (TM) drew up a data extraction table from the RevMan file that was amended by the other authors. Two authors (BG, TM) extracted data and cross-checked this for accuracy against the original publications. Errors were corrected where necessary. Where the treatment regimen was not published, one author (BG) attempted to contact the authors. If the authors were unable to be contacted, the study was excluded.

### Assessment for risk of bias

Assessment for risk of bias was carried out by the authors of the Cochrane review in accordance with the *Cochrane Handbook for Systematic Reviews of Interventions* [9]. Full details are available within the original analysis [2].

### Statistical analysis

Network plots were created for each outcome to illustrate which interventions had been directly compared within trials. The thickness of the nodes and edges in each network is proportional to the number of patients allocated to each intervention and contributing to each pairwise comparison, respectively. An NMA was undertaken to combine results of all comparisons among interventions in a single analysis. This approach makes use of both the direct comparisons available within trials and the indirect comparisons of interventions that can be made across trials when they use a common comparator intervention [10]. All analyses were conducted using a frequentist approach assuming a random-effects model, with an equal heterogeneity variance assumed for all comparisons, using the *network* suite of Stata commands, programmed by Ian White [11]. We intended to rank the interventions according to their probability to be best, second best, third best and so forth for the different outcomes. If there had been both direct evidence and indirect evidence for any particular pairwise comparison, we would have examined their agreement using methods to examine inconsistency in NMA; in practice, we did not encounter any instances of this.

## Results

### Study characteristics

**Fig. 1:**
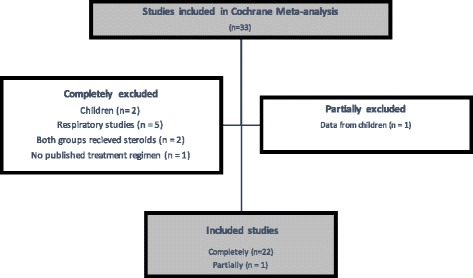
Flowchart for included and excluded studies

Thirty-three studies were included by the authors of the Cochrane review (see Additional file 1: Tables S1 and S2). Eleven studies were excluded from our analyses. Five were excluded because the study was designed to look at respiratory function in pneumonia only, not sepsis [12–16]. Two studies were excluded because they used children as their study population [17, 18]. We excluded data from children in one study, but included the data from adults [19]. Two studies were excluded because both intervention groups received steroids (as a cross-over study [20] or for different durations [21]). One study was excluded because no information regarding the treatment regimens used in the study was available [22]. The flowchart for study inclusion and exclusion is presented in Fig. 1.

### NMA results

Most CIs are very wide and often include the null value, both at the study level (see Additional file 2: Figures S1–S7) and at the NMA level. For that reason, rankings of treatments may be misleading for this review and hence are not included. Conclusions for each outcome are described below.

#### Mortality up to 28 days

**Fig. 2:**
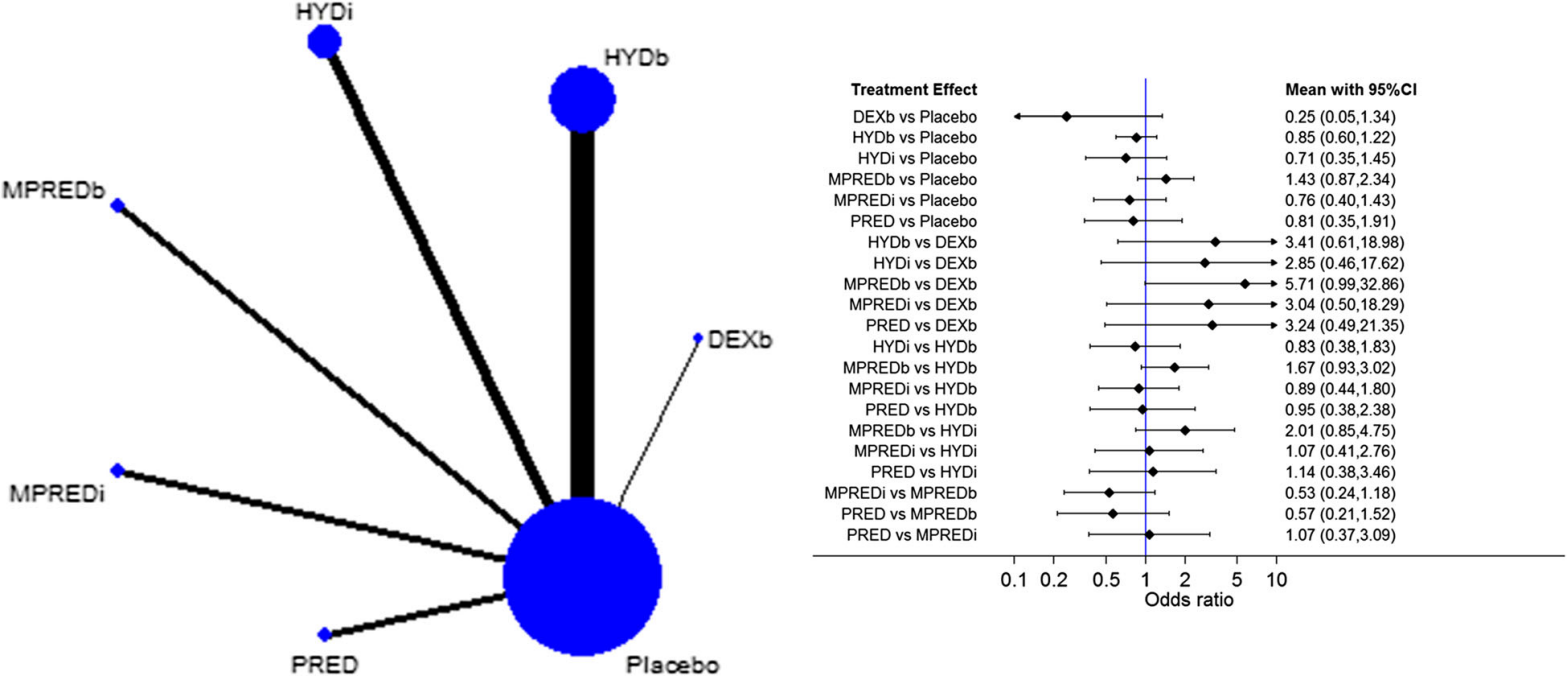
Network plot (*left*) and network meta-analysis results (*right*) of mortality up to 28 days for the different interventions. ORs <1 favour the first intervention. *DEXb* Dexamethasone bolus, *HYDb* Hydrocortisone bolus, *HYDi* Hydrocortisone infusion, *MPREDb* Methylprednisolone bolus, *MPREDi* Methylprednisolone infusion, *PRED* Prednisolone

There is evidence that boluses of methylprednisolone increase the risk of mortality compared with boluses of dexamethasone (OR 5.71, 95% CI 0.99–32.9), and there is weak evidence that boluses of dexamethasone reduce the risk of mortality at 28 days compared with placebo (OR 0.25, 95% CI 0.05–1.34) (see Fig. 2 and in Additional file 2: Figure S1). The results showed the same trends (with some loss of precision) after excluding 14-day mortality.

#### Hospital mortality

There is weak evidence that boluses of dexamethasone may reduce the risk of in-hospital mortality compared with placebo (OR 0.47, 95% CI 0.15–1.46) (see Additional file 2: Figures S2 and S8). The results suggest that infusions of hydrocortisone might increase the risk compared with boluses of dexamethasone (OR 3.06, 95% CI 0.72–12.9) and compared with boluses of hydrocortisone (OR 2.00, 95% CI 0.72–5.59).

#### Incidence of gastrointestinal bleeding

The results for incidence of GI bleeding do not enable clear conclusions to be drawn (see Additional file 2: Figures S6 and S9).

#### Incidence of superinfections

There is weak evidence that boluses of dexamethasone may increase the risk of superinfections compared with placebo (OR 2.78, 95% CI 0.73–10.6) (see Additional file 2: Figures S7 and S10). There is some evidence that both boluses (OR 0.32, 95% CI 0.09–1.19) and continuous infusions of methylprednisolone (0.23, 95% CI 0.05–1.08) may reduce this risk compared with boluses of dexamethasone.

#### Shock reversal

**Fig. 3:**
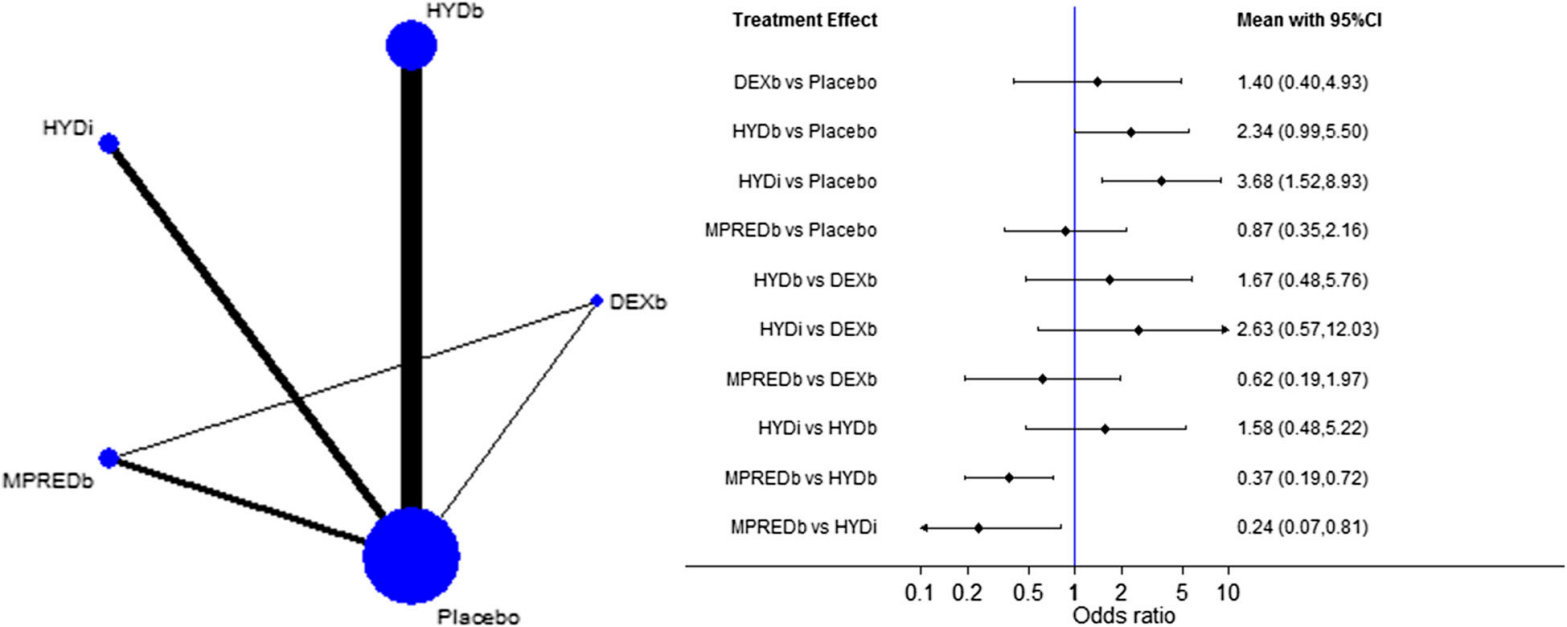
Network plot (*left*) and network meta-analysis results (*right*) of the incidence of shock reversal for the different interventions. ORs >1 favour the second intervention. *DEXb* Dexamethasone bolus, *HYDb* Hydrocortisone bolus, *HYDi* Hydrocortisone infusion, *MPREDb* Methylprednisolone bolus, *MPREDi* Methylprednisolone infusion, *PRED* Prednisolone

There is strong evidence that boluses of methylprednisolone are less likely to result in shock reversal than hydrocortisone boluses (OR 0.37, 95% CI 0.19–0.72) and infusions (OR 0.24, 95% CI 0.07–0.81) (see Fig. 3 and Additional file 2: Figure S5). There is also evidence that hydrocortisone increases the likelihood of shock reversal compared with placebo when given as a bolus (OR 2.34, 95% CI 0.99–5.50) or as an infusion (OR 3.68, 95% CI 1.52–8.93).

#### ICU mortality

Only a small subset of the database provided information for ICU mortality, so NMA for this outcome was not performed. However, one of the studies provides evidence that an infusion of methylprednisolone reduces the risk of this outcome compared with placebo (OR 0.32, 95% CI 0.10–0.99) (see Additional file 2: Figure S3).

#### Hospital LoS

Only a small subset of the studies in the database provided information for hospital LoS; thus, NMA for this outcome was not performed. The results do not enable clear conclusions to be drawn.

#### ICU LoS

Only a small subset of the database provided information for ICU LoS; thus, NMA was not performed for this outcome. However, results from two studies suggest that hydrocortisone infusion may reduce the ICU LoS compared with placebo. One of the studies provided strong evidence supporting this hypothesis (mean difference of 7 days, 95% CI 2.35 to 11.7 days), whereas the other study yielded a less precise result (mean difference of 9 days, 95% CI −3.64 to 21.6 days).

## Discussion

This NMA has provided no clear evidence that any one intervention or treatment regimen is better than any other across the spectrum of outcomes. The use of glucocorticoids in the critically ill is commonplace, but its targets and true indications are hazy. There have been three indications for the use of glucocorticoids in sepsis over the last century. First, they were given as immunosuppressants; large doses of glucocorticoids that have significant immune effects such as dexamethasone and methylprednisolone were given until studies in the late 1980s [23–27] showed a trend towards increased rates of superinfection and these high dose studies were halted. Second, there have been attempts to identify and treat a group of patients who are thought to be relatively deficient in glucocorticoids during their critical illness (critical illness-related corticosteroid insufficiency [1]), although sensitive and specific diagnostic tests for this remain to be found [28–31]. Last, and more recently, there has been a movement towards giving all patients who are unresponsive to, or on high-dose vasopressors, so called low-dose hydrocortisone (<400 mg/day) [32–34], in effect using it as a non-catecholamine vasoconstrictor. It is this approach that is currently recommended in the Surviving Sepsis guidelines [35]. This variation of study design intention has led to significant heterogeneity between the studies in this area. To try and reduce this, we excluded studies specifically designed with respiratory function as the outcome [12–16]. The hypothalamic-pituitary-adrenal axis of children is different from that of adults and develops across childhood and puberty [36]. Including the outcomes of children would further increase heterogeneity if assimilated with adult studies, and therefore data from children were also excluded for this analysis. In health [6], major surgery [7] and critical illness [8], adrenocorticotropic hormone (ACTH) and cortisol are secreted in a pulsatile manner generated by the positive feedforward and negative feedback of these two hormones. This pulsatility is critical for patients with absolute cortisol deficiency [37, 38] to maintain normal glucocorticoid receptor (GR) signalling and optimal physiological function. The pulsatile secretion pattern also translates into the pattern of receptor binding [39], with continuous and intermittent binding yielding both quantitative and qualitative differences in gene transcription [40]. In health, much of the effect of glucocorticoids is mediated by the mineralocorticoid receptor, whereas at higher (stress) levels, the effects are mediated mainly through the GR. This, coupled with the knowledge that cortisol binding globulin is saturable [41] at plasma cortisol levels of around 400 nmol/L, means that GR may be continually activated during critical illness rather than the intermittent activation of health. Intermittent delivery of hydrocortisone under these conditions may not yield the benefits seen under non-stressed conditions. There is some early animal evidence that there are no pattern dependent effects (continuous versus pulsatile) seen at glucocorticoid-responsive genes when high-dose plasma corticosteroids are used [42]. More studies are required to clarify the differential effects of these different glucocorticoid patterns.

The drive behind this NMA was to gain information that may aid in the design of improved treatment regimens. This analysis shows that hydrocortisone is likely to lead to shock reversal, but that this does not yet translate into improved mortality outcomes. Design of studies in this area should now be focused on elucidating the optimal dose and regimen for glucocorticoid treatment using hydrocortisone, yielding the benefits of improved cardiovascular reactivity and capillary permeability, with the lowest risk of hyperglycaemia, superinfection and GI bleeding.

### Comparison with other studies

There have been previous meta-analyses of steroids within the critical care environment [2–4], but, to our knowledge, this is the first using an NMA approach and the first analysis focussing on the effect of the therapeutic agent and regimen. Although all corticosteroids possess the immune, metabolic and fluid homeostatic features of their group, marked differences in the activity of each drug in these features exists. Authors of the previous meta-analyses have always used pairwise techniques to compare steroids versus placebo. In two cases, this was using a frequentist approach [2, 4]. In the third case, the different effects of high-dose (>1000 mg/day) versus low-dose (<1000 mg/day) hydrocortisone equivalents in synthetic ACTH responders and non-responders were analysed using a Bayesian approach [3]. The results of all three were also inconclusive in terms of mortality, as our analysis is. The Bayesian meta-analysis, like our analysis, did show a high probability of treatment efficacy of low-dose hydrocortisone treatment in terms of shock reversal. This again was not at the expense of higher complications, and this pattern persists regardless of the method of analysis [2–4].

### Strengths and limitations

The strengths of this study are that it takes the robust, structured approach of a Cochrane review and applies a different statistical approach to the data. NMA allows multiple pairwise comparisons and therefore may allow a more granular approach to answering the clinical question than a generic ‘steroids versus placebo’ question. In principle, for a network analysis to hold true, the interventions should be ‘jointly randomisable’. That is, a participant in any trial meeting the inclusion criteria could have been randomised to any of the interventions [43]. This is true for this analysis and the underlying principle regarding why patients included in the trials designed to look at respiratory function and children were excluded. The study included data from over 3000 patients, and therefore it may appear that its ability to discriminate small differences in outcome should be reasonable. However, this ability is significantly reduced by dividing the patients into multiple treatment groups, whereby the number of patients in each group is fewer.

The limitations of the study include that there were few direct comparisons of treatment regimens (all but two studies were intervention versus placebo). This means that the strength of inference made in an NMA between different interventions is not as robust as it could be and that consistency between direct and indirect evidence could not be assessed. This study used data from the last 50 years. Improvements in intensive care over the last half-century are myriad, and the breadth of patients, in terms of both age and co-morbidities now treated within a critical care environment, has increased. This means that bias will have inevitably crept in. Whilst the inclusion criteria for studies may not have changed, the population from which they are drawn will. Many patients not considered appropriate for critical care in the 1970s and 1980s are the mainstay of work for many critical care units in developed countries [44]. As mentioned previously, the glucocorticoids used in trials have changed with time from high-dose immunosuppressive agents towards lower-dose hydrocortisone. Thus, although at an individual level the included patients are ‘jointly randomisable’, between the groups there are likely to be significant differences. We also did not compare the effect of the dose of corticosteroids. The network is already rather sparse, and therefore our ability to differentiate between different doses and different agents is very low. Other limitations included that many of the studies reported different outcome measures for the same headline outcome. This made performing NMA on these outcomes impossible in many circumstances. This was most striking for ‘hyperglycaemia’, where the definition varied between ‘total insulin dose’ [32] blood glucose >150 mg/dl on any day [45], an increase of >200 mg/dl [46] and others, hence its exclusion as an outcome. Standardisation of outcome definitions for critical care trials would improve this problem going forward.

## Conclusions

This NMA has provided no clear evidence that any one treatment regimen of glucocorticoids is more likely to be effective than any other in the treatment of septic shock. This was seen across the outcome measures. There is good evidence that both hydrocortisone boluses and infusions increased the likelihood of shock reversal compared with placebo and boluses of methylprednisolone. Current guidelines for the management of sepsis [35] reflect this. Physiological patterns of hydrocortisone in both blood and at the level of GRs in the tissues are pulsatile, and at physiological levels, these patterns change both the quantity and the type of genomic outputs. Going forward, studies of steroids in sepsis should be focused on the most appropriate dose of hydrocortisone delivered in the correct pattern. Definitions of outcome within the critical care literature vary widely, and the case should be made for standardisation.

## Additional files

Additional file 1: Summary tables of included and excluded study characteristics [47–60]. (DOCX 87 kb)

Additional file 2: Pairwise comparisons of the individual studies, NMA plots and results for those outcomes not included in the main text. (DOCX 2566 kb)

## Abbreviations

ACTH: Adrenocorticotropic hormone
ARDS: Acute respiratory distress syndrome
GI: Gastrointestinal
GR: Glucocorticoid receptor
ICU: Intensive care unit
LoS: Length of stay
NMA: Network meta-analysis
DEXb: Dexamethasone bolus
HYDb: Hydrocortisone bolus
HYDi: Hydrocortisone infusion
MPREDb: Methylprednisolone bolus
MPREDi: Methylprednisolone infusion
PRED: Prednisolone
