# Supplementary material for: Corticosteroids in septic shock: a systematic review and network meta-analysis
Source: Crit Care. 2017 Mar 28;21:78. doi: 10.1186/s13054-017-1659-4 (PMC5371269; doi:10.1186/s13054-017-1659-4)
Supplement: Supplementary file 1 — Summary tables of included and excluded study characteristics [47–60]. (DOCX 87 kb) [file 13054_2017_1659_MOESM1_ESM.docx]

| **Study, Year** | **Bolus / Infusion** | **Location(s)** | **Design** | **Participants** | **Intervention** | **Comparator** | **Primary Outcomes** | **Secondary Outcomes** |
| --- | --- | --- | --- | --- | --- | --- | --- | --- |
| Annane 2002[47] | Bolus | France | Multi-centre (19)  RCT | Adults (n=300)  Vasopressor AND Ventilator dependent septic shock.  Classified by response to ST:  Responders >9mcg/dL  Non-responders ≤9mcg/dL | Hydrocortisone 50mg Every 6hrs  AND Fludrocortisone 50mcg Every 24hrs  Duration 7 days | Placebo | 28-Day Mortality in non-responders | 28-Day Mortality in responders  ICU Mortality  Hospital Mortality  1-Year Mortality  Shock Reversal  Organ system failure free days  ICU LoS  Hospital LoS  Safety Outcomes |
| Annane 2010[32] | Bolus | France | Multi-centre (11)  RCT | Adults (n=509)  Vasopressor dependent septic shock | Hydrocortisone 50mg Every 6hrs  AND Fludrocortisone 50mcg Every 24hrs  AND IV Insulin maintaining blood glucose 80 – 110mg/dL  Hydrocortisone 50mg Every 6hrs  AND IV Insulin maintaining blood glucose 80 – 110mg/dL  Hydrocortisone 50mg Every 6hrs  AND Fludrocortisone 50mcg Every 24hrs  AND Standard blood glucose control  Hydrocortisone 50mg Every 6hrs  AND standard blood glucose control  Duration: 7 days  . | Each intervention compared with the others – ie no placebo | Hospital Mortality | 28-Day mortality  90-Day mortality  180-day mortality  ICU mortality  Vasopressor free days  Organ-failure free days  ICU LoS  Hospital LoS  Safety outcomes |
| Arabi 2011[48] | Bolus | Saudi Arabia | Single-centre  RCT | Adults (n=75)  Liver cirrhosis and septic shock | Hydrocortisone 50mg  Every 6hrs  Duration: Until shock resolution then tapered by 10mg every 48hrs until stopped | Placebo | 28 day mortality | ICU Mortality  Hospital mortality  Shock reversal  Mechanical ventilation free days  RRT free days  ICU LoS  Hospital LoS  SOFA Score at D7  Adverse events |
| Bollaert 1998[49] | Bolus | France | Multi-centre (2)  RCT | Adults (n=41)  Vasopressor AND ventilator dependent septic shock  Classified by response to ST:  Responders ≤6mcg/dL  Non-responders >6mcg/dL | Hydrocortisone 100mg  Every 8hrs  Duration:  5 days then tapered over 6 days | Placebo | Shock reversal | 28 days mortality  ICU Mortality  Hospital mortality  Improvement in haemodynamics  ICU LoS  Hospital LoS  Safety |
| Bone 1987[25] | Bolus | USA | Multi-centre (19)  RCT | Adults (n=382)  With sepsis (n=234) OR  Septic shock (n=148) | Methylprednisolone 30mg/kg  Every 6 hours  Duration: 24hrs | Placebo | For sepsis: 14 day development of shock  For septic shock: shock reversal  14 day mortality  14 day safety | None |
| Briegel 1999[50] | Infusion | Germany | Single-centre  RCT | Adults (n=40)  Vasopressor AND Ventilator dependent septic shock | Hydrocortisone 100mg loading  0.18mg/kg/hr maintainance  Duration:  Until shock reversal, then tapered off | Placebo | Shock reversal | 28 day mortality  ICU mortality  Hospital mortality  Improvement in haemodynamics  SOFA score at D7  ICU LoS  Safety |
| Chawla 1999[51] | Bolus | USA | Single-centre  RCT | Adults (n=44)  Vasopressor dependent shock | Hydrocortisone 100mg  Every 8hrs  Duration: 3 days then tapered over 4 days | Placebo | Shock reversal | 28 day mortality  Hospital mortality  Improvement in haemodynamics  ICU LoS  Safety |
| Cicarelli 2007[52] | Bolus | Brazil | Single-centre  RCT | Adults (n=29)  Vasopressor dependent septic shock | Dexamethasone 0.2mg/kg  Every 36hrs  Duration: 3 doses | Placebo | 28 day mortality  Duration of vasopressor support  Duration of mechanical ventilation |  |
| CSG 1963[19] | Infusion | USA | Multi-centre (5)  RCT | Adults (n=194)  Children (n=135)  Vasopressor dependent septic shock | Hydrocortisone  300mg for 24hrs  Tapered by 50mg per day.  PO from D3  Duration: 6 days | Placebo | Hospital mortality | Safety |
| Gordon 2014[33] | Bolus | UK | Multi-centre (4)  RCT | Adults (n=61)  Septic shock treated with vasopressin | Hydrocortisone 50mg  Every 6 hours for 5 days  Every 13hrs for 3 days  Every 24hrs for 3 days | Placebo | Plasma vasopressin concentration | Vasopressin requirements  28 day mortality  ICU mortality  Hospital mortality  Organ failure free days until D28  Shock reversal  ICU LoS  Hospital LoS  Safety |
| Hu 2009[53] | Bolus | China | Single-centre  RCT | Adults (n=77)  Septic shock | Hydrocortisone 50mg every 6hrs for 7 days  50mg every 8hrs for 3 days  50mg every 12hrs for 2 days  50mg every 24hrs for 2 days | Unknown | Time on noradrenaline  Lactate clearance | ICU Mortality  ICU LoS  Shock reversal |
| Liu 2012[54] | Bolus | China | Single-centre  RCT | Adults (n=26)  ARDS and sepsis including septic shock | Hydrocortisone 100mg  Every 8hrs  Duration:  7 days | Placebo | Unclear | 28 Day mortality  Prevalence of shock within 28days  SOFA Score  ICU LoS  Safety |
| Luce 1988[23] | Bolus | USA | Single-centre  RCT | Adults (n=75)  Sepsis AND septic shock | Methylprednisolone  30mg/kg every 6hrs  Duration: 1 day | Placebo | Prevention of ARDS | Hospital mortality  Immunological |
| Meduri 2007[55] | Infusion | USA | Multi-centre (5)  RCT | Adults (n=91)  Early ARDS  Sepsis / septic shock (n=61) – only septic shock included  Stratified by ST:  Responders (≤9mcg/dl)  Non-responders (>9mcg/dL) | Methylprednisolone Loading: 1mg/kg  Maint: 1mg/kg/d D1 – 14  0.5mg/kg/d D15-21  0.25mg/kg/d D22-25  0.125mg/kg D26-28 | Placebo | Improvement in lung injury score | Mechanical ventilation free days  MOD score at D7  28 day mortality  ICU mortality  Hospital mortality  ICU LoS  CRP on D7  Safety |
| Oppert 2005[56] | Infusion | Germany | Single-centre  RCT | Adults (n=40)  Vasopressor dependent septic shock | Hydrocortisone  Load: 50mg  Maint: 0.18mg/kg/hr  Duration: until stopping vasopressor  0.06mg/kg/hr for 1 day then reduced by 0.02mg/kg/hr every day | Placebo | Time to stopping vasopressor support | Inflammatory markers  28 day survival  SOFA score |
| Rinaldi 2006[57] | Infusion | Italy | Single-centre  RCT | Adults (n=40)  Sepsis, but no vasopressor | Hydrocortisone 300mg per day  Duration: 6days then taper | Standard care | Micro-albuminuria: creatinine ratio | Inflammatory markers  Duration of mechanical ventilation  SOFA score |
| Sabry 2011[34] | Infusion | Egypt | Multi-centre (3)  RCT | Adults (n=80)  Pneumonia and sepsis | Hydrocortisone Load: 200mg  Maint: 12.5mg/h  Duration: 7 days | Placebo | Improvement in Pa02:Fi02 ratio | SOFA score D8  Development of delayed septic shock  ICU Mortality rate |
| Schumer 1976[58] | Bolus | USA | Single-centre  RCT (3 groups) | Adults (n=172)  Septic shock and positive blood cultures | Dexamethasone (3mg/kg)  Methyprednisolone (30mg/kg) | Placebo | Hospital mortality | Complication rates |
| Sprung 1984[46] | Bolus | USA | Multi-centre (2)  RCT (4 groups) | Adults (n=59)  Vasopressor dependent shock | Dexamethasone 6mg/kg  Methylprednisolone 30mg/kg  Doses could be repeated | Standard care  Placebo | Hospital mortality  Shock reversal | Complications of septic shock  Safety |
| Sprung 2008[45] | Bolus | Europe  Israel | Multi-centre (52)  RCT | Adults (n=499)  Septic shock | Hydrocortisone  50mg every 6hrs – 5 days  50mg every 12hrs – 3 days  50mg every day – 3days | Placebo | 28 day mortality in non-responders | 28 day mortality in all  ICU mortality  Hospital mortality  1 year mortality  Shock reversal  Organ system failure free days  Safety |
| VASSCSG 1987[24] | Infusion | USA | Multi-centre(10)  RCT | Adults (n=223)  Septic shock (n=100) | Methylprednisolone  Load 30mg/kg  Maint: 5mg/kg/hr  Duration 9hrs | Placebo | 14 day mortality | Complications |
| Yildiz 2002[59] | Bolus | Turkey | Single-centre  RCT | Adults (n=40)  Sepsis (n=14)  Septic shock (n=9) | Prednisolone  5mg@ 0600  [2.5mg@1800](mailto:2.5mg@1800)  Duration: 10 days | Placebo | 28 days mortality | Hospital mortality  Safety |
| Yildiz 2011[60] | Bolus | Turkey | Single-centre  RCT | Adults (n=55)  Sepsis OR septic shock | Prednisolone  10mg@0600  5mg @ 1400 and 2200 | Placebo | 28 day mortality | Reversal of organ failure  LoS  Safety |

**Table 1**. Summary of included study characteristics. Studies with some data excluded from this analysis are shaded in grey

| **Study, Year** | **Bolus / Infusion** | **Location(s)** | **Design** | **Participants** | **Intervention** | **Comparator** | **Primary Outcomes** | **Secondary Outcomes** |
| --- | --- | --- | --- | --- | --- | --- | --- | --- |
| Confalonieri 2005[12] | Infusion | Italy | Multi-centre (6)  RCT | Adults (n=46)  Severe community acquired pneumonia | Hydrocortisone 200mg loading  10mg/hr maintenance  Duration:  7 days then tapered over 4 days | Placebo | Improvement in Pa02:Fi02 ratio and MODS score by D8 | Duration of mechanical ventilation  60 day mortality  ICU mortality  Hospital mortality  ICU LoS  Hospital LoS  Safety |
| Huh 2006[20] | Bolus | South Korea | Single-centre  RCT | Adults (n=82)  Septic shock AND adrenal insufficiency | Hydrocortisone 50mg  Every 6hrs  Duration:  One group 7 days, one group 3 days | Direct comparison of interventions | 28 day mortality | ICU Mortality  Hospital mortality  ICU LoS  Hospital LoS  Shock reversal  Duration of mechanical ventilation  Safety |
| Keh 2003[21] | Infusion | Germany | Single-centre  Randomised controlled cross over trial | Adults (n=40)  Vasopressor dependent septic shock | Hydrocortisone 100mg followed by infusion 10mg/hr for 3 days | Placebo  X-over design – both groups got both treatments | Immune response | Improvement in haemodynamics  Organ system failure |
| Meijvis 2011[13] | Bolus | Netherlands | Multi-centre (2)  RCT | Adults (n=304)  CAP presenting to ED | Dexamethasone 5mg every 24hrs  Duration: 4 days | Placebo | Hospital LoS | 30 days mortality  Hospital mortality  IV ABx Rx duration  ICU Admission  Inflammation markers  Health performance  Lung function  Safety |
| Rezk 2013[14] | Infusion | Egypt | Single-centre  RCT | Adults (n=40)  ARDS and pneumonia | Methylprednisolone  1mg/kg/d D1-D14  0.5mg/kg/d D15-D21  0.25mg/kg/d D22-25  0.125mg/kg/d D26-28 | Placebo | Unclear | Short-term mortality (what?)  Time on mechanical ventilation  Vital signs  Safety |
| Slusher 1996[17] | Bolus | Kenya, Nigeria | Multi-centra (2)  RCT | Children (n=17)  Sepsis or septic shock | Dexamethasone  0.20mg/kg every 8 hrs  Duration 2 Days | Placebo | Hospital mortality | Haemodynamic stability after 48hrs  Complications |
| Snijders 2010[15] | Bolus | Netherlands | Single-centre  RCT | Adults (n=213)  Pneumonia | Prednisolone 40mg every day  Duration: 7 days | Placebo | D7 / D30 treatment failure | Time to clinical stability  Hospital LoS  30 day mortality  Inflammatory markers  Safety |
| Tandan 2005  [22] | Unknown | India | Single-centre  RCT | Adults (n=28)  Septic shock and adrenal insufficiency | Unknown | Placebo | 28 day mortality or survival to hospital discharge | Shock reversal  Imprpovement in APACHE II  Safety |
| Torres 2015[16] | Bolus | Spain | Multi-centre(3)  RCT | Adults (n=61)  Severe pneumonia and high CRP | Methylprednisolone (0.5mg/kg) every 12 hrs  Duration: 5 days | Placebo | Rate of treatment failure | Time to clinical stability  ICU LoS  Hospital LoS  Hospital mortality  Safety |
| Valoor 2009[18] | Bolus | India | Single-centre RCT | Children (n=38)  Septic shock unresponsive to fluid therapy | Hydrocortisone Load: (1.25mg/kg/d) every 6hrs  Maint: (0.625mg/kg/day) every 6 hours  Duration: 7 Days | Placebo | Time to shock reversal | Dose of vasopressor  Mortality  Safety |

**Table 2.** Studies included by the Cochrane meta-analysis and excluded by this analysis.
