# Supplementary material for: Corticosteroids in septic shock: a systematic review and network meta-analysis
Source: Crit Care. 2017 Mar 28;21:78. doi: 10.1186/s13054-017-1659-4 (PMC5371269; doi:10.1186/s13054-017-1659-4)
Supplement: Supplementary file 2 — Pairwise comparisons of the individual studies, NMA plots and results for those outcomes not included in the main text. (DOCX 2566 kb) [file 13054_2017_1659_MOESM2_ESM.docx]

**Additional file**

Figure S1. Pairwise comparison of the individual studies for the outcome - Mortality up to 28 days. DEXb: dexamethasone bolus; HYDb: hydrocortisone bolus; HYDi: hydrocortisone infusion; MPREDb: methylprednisolone bolus; MPREDi: methylprednisolone infusion; PRED: prednisolone.

Figure S2. Pairwise comparison of the individual studies for the outcome – Hospital mortality. DEXb: dexamethasone bolus; HYDb: hydrocortisone bolus; HYDi: hydrocortisone infusion; MPREDb: methylprednisolone bolus; MPREDi: methylprednisolone infusion; PRED: prednisolone.

Figure S3. Pairwise comparison of the individual studies for the outcome – ICU Mortality. HYDb: hydrocortisone bolus; HYDi: hydrocortisone infusion; MPREDi: methylprednisolone infusion;

Figure S4. Pairwise comparison of the individual studies for the outcome – ICU Length of Stay. HYDb: hydrocortisone bolus; HYDi: hydrocortisone infusion; MPREDi: methylprednisolone infusion;

Figure S5. . Pairwise comparison of the individual studies for the outcome – Shock reversal. DEXb: dexamethasone bolus; HYDb: hydrocortisone bolus; HYDi: hydrocortisone infusion; MPREDb: methylprednisolone bolus; MPREDi: methylprednisolone infusion; PRED: prednisolone.

Figure S6. Pairwise comparison of the individual studies for the outcome – Gastro-intestinal bleeding. DEXb: dexamethasone bolus; HYDb: hydrocortisone bolus; HYDi: hydrocortisone infusion; MPREDb: methylprednisolone bolus; MPREDi: methylprednisolone infusion; PRED: prednisolone.

Figure S7. Pairwise comparison of the individual studies for the outcome – Superinfections. DEXb: dexamethasone bolus; HYDb: hydrocortisone bolus; HYDi: hydrocortisone infusion; MPREDb: methylprednisolone bolus; MPREDi: methylprednisolone infusion; PRED: prednisolone.

|  | 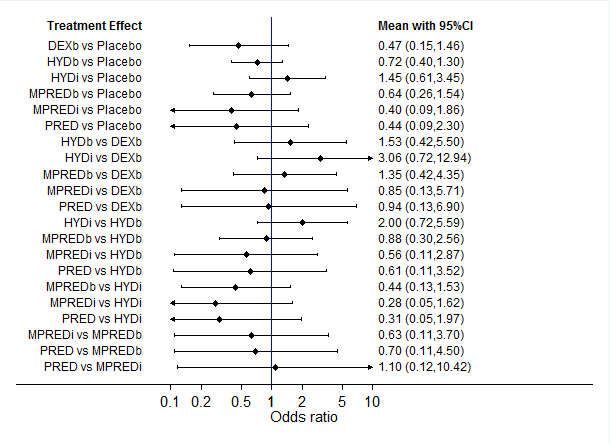 |
| --- | --- |

Figure S8. Network plot (left) and NMA results (right) of Hospital mortality for the different interventions. Odd ratios less than 1 favour the first intervention.

|  | 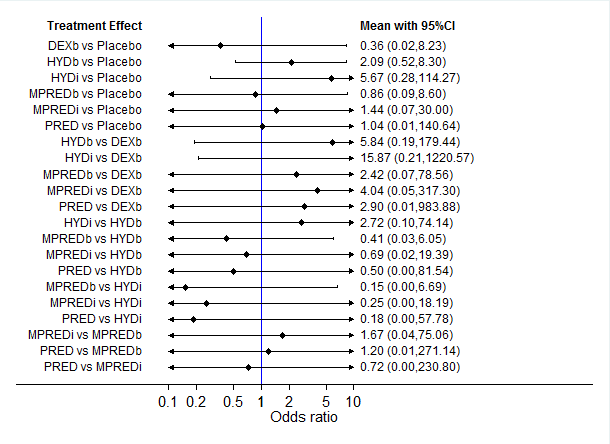 |
| --- | --- |

Figure S9. Network plot (left) and NMA results (right) for the incidence of gastro-intestinal bleeding for the different interventions. Odd ratios less than 1 favour the first intervention.

|  | 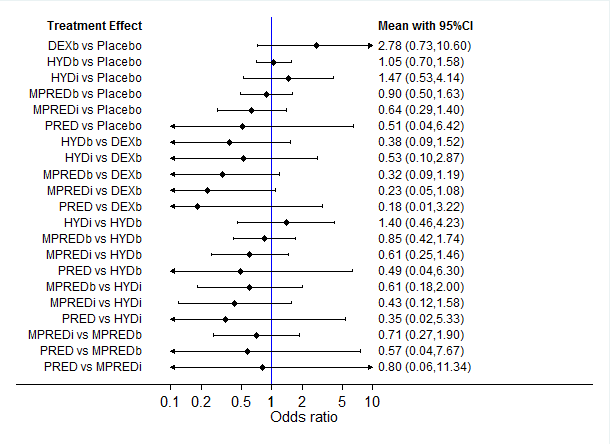 |
| --- | --- |

Figure S10. Network plot (left) and NMA results (right) for the incidence of superinfections for the different interventions. Odd ratios less than 1 favour the first intervention.
